# Effective Properties of Composites with Periodic Random Packing of Ellipsoids

**DOI:** 10.3390/ma10020112

**Published:** 2017-01-26

**Authors:** Xiaoying Zhuang, Qing Wang, Hehua Zhu

**Affiliations:** 1 Department of Geotechnical Engineering, Civil Engineering College, Tongji University, Shanghai 200092, China; myheart6144@126.com (Q.W.); zhuhehua@tongji.edu.cn (H.Z.); 2 Institute of Continuum Mechanics, Leibniz-University Hannover, 30167 Hannover, Germany; 3 State Key Laboratory for Disaster Reduction in Civil Engineering, Tongji University, Shanghai 200092, China

**Keywords:** multiscale modelling, computational homogenization, random periodic packing, molecular dynamics, composite properties

## Abstract

The aim of this paper is to evaluate the effective properties of composite materials with periodic random packing of ellipsoids of different volume fractions and aspect ratios. Therefore, we employ computational homogenization. A very efficient MD-based method is applied to generate the periodic random packing of the ellipsoids. The method is applicable even for extremely high volume fractions up to 60%. The influences of the volume fraction and aspect ratio on the effective properties of the composite materials are studied in several numerical examples.

## 1. Introduction

Composite materials are widely used in engineering applications. Extraction of the mechanical properties by experiments is often expensive, time consuming and sometimes unfeasible. Therefore, it is important to develop modelling approaches, such as computational homogenization, to extract the mechanical properties of composite materials. The mechanical properties of composite materials are strongly determined by their microstructure, e.g., the shape, size and distribution of the inclusions in the matrix. Generating complex microstructures for RVEs (representative volume elements), which are commonly used in computational homogenization, remains a challenge.

Computational multiscale methods, which are commonly used for extracting the mechanical properties of composites, can be classified into hierarchical, semi-concurrent and concurrent methods [1]. A classical hierarchical multiscale method transfers information from the fine scale to the coarse scale only. It is particularly efficient for linear materials and becomes expensive for non-linear material behavior, since all possible deformation states have to be accounted for in order to extract the material response. The Cauchy–Born rule or the FE^2^ approach [2,3] is a classical semi-concurrent method, which is also based on RVEs. However, in the FE^2^ approach, information is passed back also from the coarse scale to the fine scale in the course of a simulation. Though semi-concurrent multiscale methods are computationally expensive, they account only for deformation states that actually occur in a simulation, and the constitutive model is determined on the fly. Concurrent multiscale methods also transfer information back from the coarse scale to the fine scale. However, concurrent multiscale methods do not exploit RVEs. Instead, the fine scale is directly embedded into the coarse scale and coupling via methods such as Lagrange multipliers, etc. There are numerous concurrent multiscale methods, such as the handshake domain method [4,5,6] or interface coupling methods [7].

Homogenization methods can be classified into two categories: analytical homogenization methods [8,9,10,11,12,13,14] and computational homogenization methods [15,16,17,18,19,20,21,22]. The analytical homogenization methods, which are based on the Eshelby inclusion solution [8], are limited to linear materials and simple microstructures. In the computational homogenization methods, the macroscopic constitutive model is defined “on the fly” during the simulation. The linear [23] and nonlinear problems [3], even the multi-physics problems [24,25,26,27,28,29,30], can be addressed with these methods. The computational homogenization methods can deal with the composite materials with complex microstructures [31].

Efficient and reliable algorithms for the generation and discretization of the microstructure of composites are of major importance. The RVE generation approaches can be divided into two main categories: random sequential adsorption (RSA) and molecular dynamics (MD)-based algorithms. In the RSA algorithm [32,33,34,35,36], the positions of the inclusions are drawn in sequence, and the contact between the inclusions is checked until the number of inclusions or the volume fraction is reached. The RSA is efficient for small volume fraction (up to 30%). When the number or the volume fraction of inclusions is higher, the RSA may fail. The main idea of the MD-based algorithm [37,38,39] is as follows: All inclusions are randomly created in the unit cell. Each inclusion has a null volume and a random velocity. Then, the inclusions move, and their volumes gradually increase. When collision between two inclusions occurs, the velocities of the inclusions are updated according to the law of the conservation of momentum. The process stops when the number of inclusions or the volume fraction is reached. The MD-based algorithm is more efficient for higher volume fractions. It was even possible to reach a volume fraction of 70% [40]. Although the methods in [41] can generate more inclusion shapes, such as “block-like” shapes, it also needs more iterations to reach the best design. The MD-based algorithm will be more efficient than the genetic search method for dense packing inclusions. Due to the collision detection between the “block-like” shapes being more complex, only the ellipsoids are considered in this paper.

The content of the paper is outlined as follows. In Section 2, the computational homogenization model is described. The MD-based RVE generation approaches are detailed in Section 3. Section 4 presents the analysis of the effective properties of composite materials depending on the microstructure of the RVE. The paper is concluded in Section 5 with suggested topics for future studies.

## 2. Computational Homogenization Method for Composite Material

### 2.1. Governing Equations

The computational homogenization method has two assumptions [23]. The first assumption is the separation of the length scales into micro- and macro-scales. The microscale length is several magnitudes smaller than the macroscale length, so that the microscopic heterogeneities and the macroscopic homogeneities can be reflected. Therefore,

(1) $$l_{micro}\ll l_{macro},$$

where $l_{micro}$, $l_{macro}$ are the length of the micro- and macro-scales, respectively.

The second assumption is the periodicity of the microstructure. Two different scales ***x*** and ***y***, associated respectively with the macroscale and microscale domains, are assumed. The relation between the microscale and macroscale spatial coordinates is:

(2) $$y_{i}=\frac{x_{i}}{\varepsilon },$$

where $x_{i}$ (*i* = 1, 2, 3) denotes the component of macroscale coordinates; $y_{i}$ corresponds to the microscale coordinates; ε≪1 is a scale factor between the macroscale and the microscale.

Consider a macroscale domain $\Omega^{\varepsilon }$ with boundary $\Gamma^{\varepsilon }$, which has a microstructure associated with a microscale domain denoted as Θ. The governing equation of the linear elastic problem may be expressed as:

(3) $$\sigma_{ij,x_{j}}^{\varepsilon }+b_{i}=0\text{ in }\Omega^{\varepsilon }$$

(4) $$\sigma_{ij}^{\varepsilon }=L_{ijmn}^{\varepsilon }e_{mn}^{\varepsilon }\text{ in }\Omega^{\varepsilon }$$

(5) $$e_{mn}^{\varepsilon }=\frac{1}{2}(u_{m,x_{n}}^{\varepsilon }+u_{n,x_{m}}^{\varepsilon })\text{ in }\Omega^{\varepsilon }$$

The displacement and traction boundary conditions applied to the domain are:

(6) $$u_{i}^{\varepsilon }=\bar{u_{i}}\text{ in }\Gamma_{u}^{\varepsilon }$$

(7) $$\sigma_{ij}^{\varepsilon }n_{j}=\bar{t_{i}}\text{ in }\Gamma_{t}^{\varepsilon }$$

where the subscript i,j,m,n∈{1,2,3}, $\sigma_{ij}^{\varepsilon }$, $e_{mn}^{\varepsilon }$, $u_{i}^{\varepsilon }$, $L_{ijmn}^{\varepsilon }$ are the components of stress, strain, displacement and constitutive tensor, respectively, $b_{i}$ is the body force vector, $\bar{u_{i}}$, $\bar{t_{i}}$ are the prescribed displacement and traction, $\Gamma_{u}^{\varepsilon }$, $\Gamma_{t}^{\varepsilon }$ are the displacement and traction boundary and $n_{j}$ is the outward normal vector of the boundary. The subscripts $x_{i}$, $y_{i}$ refer to the spatial coordinates of the macroscale and microscale, respectively.

### 2.2. The Asymptotic Expansion of Displacement

Assuming the material responses at the macroscale model denoted as ***x*** and the microscale model denoted as ***y*** are related, the displacement, strain and stress fields can be approximated by using the asymptotic expansions [23] in terms of ε, the scale factor between the micro- and macro-scales,

(8) $$u_{i}^{\varepsilon }(x)=\varepsilon^{0}u_{i}^{\left(0\right)}(x,y)+\varepsilon^{1}u_{i}^{\left(1\right)}(x,y)+\varepsilon^{2}u_{i}^{\left(2\right)}(x,y)+ο(\varepsilon^{2})$$

(9) $$e_{ij}^{\varepsilon }=\varepsilon^{-1}e_{ij}^{\left(-1\right)}+\varepsilon^{0}e_{ij}^{\left(0\right)}+\varepsilon^{1}e_{ij}^{\left(1\right)}+ο(\varepsilon )$$

(10) $$\sigma_{ij}^{\varepsilon }=\varepsilon^{-1}\sigma_{ij}^{\left(-1\right)}+\varepsilon^{0}\sigma_{ij}^{\left(0\right)}+\varepsilon^{1}\sigma_{ij}^{\left(1\right)}+ο(\varepsilon )$$

where $u_{i}^{\left(r\right)}$ is the *y*-periodic function of the order *r* of the displacement field. Substituting the asymptotic expansion of the displacements in Equation (8) into the strain-displacement relations in Equation (5), regrouping the terms according to the Equation (9), then the strain terms can be derived as follows,

(11) $$e_{ij}^{\left(-1\right)}=\frac{1}{2}\left(\frac{\partial u_{i}^{\left(0\right)}}{\partial y_{j}}+\frac{\partial u_{j}^{\left(0\right)}}{\partial y_{i}}\right)$$

(12) $$e_{ij}^{\left(0\right)}=\frac{1}{2}\left(\frac{\partial u_{i}^{\left(0\right)}}{\partial x_{j}}+\frac{\partial u_{j}^{\left(0\right)}}{\partial x_{i}}+\frac{\partial u_{i}^{\left(1\right)}}{\partial y_{j}}+\frac{\partial u_{j}^{\left(1\right)}}{\partial y_{i}}\right)$$

(13) $$e_{ij}^{\left(1\right)}=\frac{1}{2}\left(\frac{\partial u_{i}^{\left(1\right)}}{\partial x_{j}}+\frac{\partial u_{j}^{\left(1\right)}}{\partial x_{i}}+\frac{\partial u_{i}^{\left(2\right)}}{\partial y_{j}}+\frac{\partial u_{j}^{\left(2\right)}}{\partial y_{i}}\right)$$

By substituting the strain in Equation (9) into the constitutive Equation (4), we have the asymptotic expansion of the stress as:

(14) $$\sigma_{ij}^{\left(r\right)}=L_{ijmn}e_{mn}^{\left(r\right)}\;r=-1,\;0,\;1$$

The asymptotic expansion of the equilibrium equation can be obtained by substituting the stress in Equation (10) into the equilibrium Equation (3) and regrouping the terms according to the order of ε:

(15) $$\varepsilon^{-2}\to \frac{\partial \sigma_{ij}^{\left(-1\right)}}{\partial y_{j}}=0$$

(16) $$\varepsilon^{-1}\to \frac{\partial \sigma_{ij}^{\left(-1\right)}}{\partial x_{j}}+\frac{\partial \sigma_{ij}^{\left(0\right)}}{\partial y_{j}}=0$$

(17) $$\varepsilon^{0}\to \frac{\partial \sigma_{ij}^{\left(0\right)}}{\partial x_{j}}+\frac{\partial \sigma_{ij}^{\left(1\right)}}{\partial y_{j}}+\Delta b_{i}=0$$

After obtaining the equilibrium equations and boundary conditions related to different orders of ε, the differential equations of the two-scale problems can be derived by solving Equations (15)–(17). After solving the equations at different scales, the multiscale problem is decomposed into:

The macroscale problem:

(18) $$\begin{array}{cc} \frac{\partial }{\partial x_{j}}\left({\bar{L}}_{ijkl}e_{kl}^{\left(0\right)}\right)+{\langle b_{i}\rangle }_{y}=0 & \Omega \\ u_{i}^{\left(0\right)}={\bar{u}}_{i} & \Gamma_{u}\\ {\bar{L}}_{ijkl}e_{kl}^{\left(0\right)}n_{j}={\bar{t}}_{i} & \Gamma_{t} \end{array},$$

The microscale problem:

(19) $$\begin{array}{cc} \frac{\partial }{\partial y_{j}}\left(L_{ijmn}\left(I_{mn}^{kl}+\frac{\partial \chi_{m}^{kl}}{\partial y_{n}}\right)\right)=0 & \Theta \\ \chi_{i}^{mn}(y)=0 & \Gamma_{y}^{vert}\\ \chi_{i}^{mn}(y+\Theta )=\chi_{i}^{mn}(y) & \Gamma_{y} \end{array}$$

and the bridging between the two scales is given by:

(20) $${\bar{L}}_{ijkl}={\langle \sigma_{ij}^{kl}\rangle }_{y}=\frac{1}{\left|\Theta \right|}\int_{\Theta }L_{ijmn}\left(I_{mn}^{kl}+\frac{\partial \chi_{m}^{kl}}{\partial y_{n}}\right)d\Theta$$

**Figure 1:**
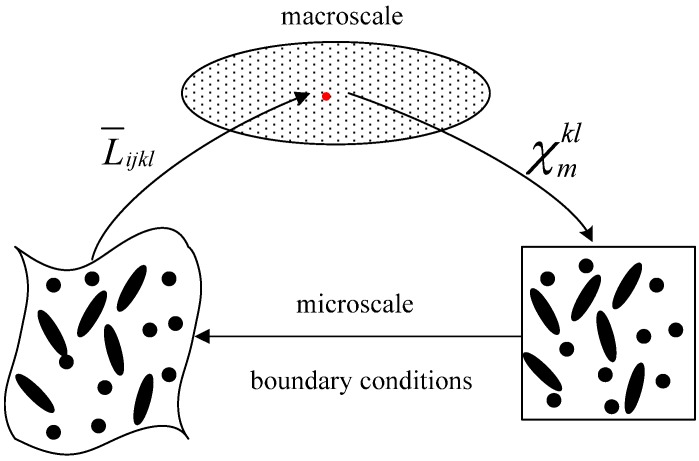
Computational homogenization schemes.

The computational homogenization method may be divided into four steps [42] as illustrated in Figure 1, which has been previously implemented with convergence studies in ABAQUS [23]:

Step 1. Solve the microscale problem in Equation (19) with six unit right-hand side strain vectors, and compute the stress $\sigma_{ij}^{kl}$ with the associated strain.
Step 2. Compute the homogenized elastic tensor ${\bar{L}}_{ijkl}$ by Equation (20).
Step 3. Solve the macroscale problem in Equation (18) with the homogenized elastic tensor.
Step 4. Post-process the local stress of the critical RVE.

## 3. RVE Generation

### 3.1. RVE Generation Algorithm

**Figure 2:**
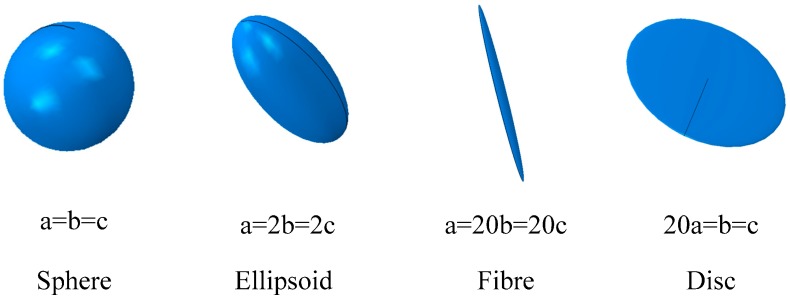
Ellipsoids with different aspect ratios.

The shapes of the inclusions in the microstructure are various, and they affect the effective properties of random two-phase materials [43]. It is hard to describe the shape precisely and improve the computational efficiency at the same time. The ellipsoids with different aspect ratios can describe a wide range of shapes; see Figure 2. The sphere and normal ellipsoid can simulate ‘regular’ reinforcements. Fiber reinforcements may be simulated by prolate ellipsoids. Joints in the rock mass, microcrack or two-dimensional reinforcement, such as graphene, are usually described as discs, so they can be simulated by the oblate ellipsoids. In this paper, the generation algorithm of periodic random packing of ellipsoids will be presented, which is a molecular dynamics (MD)-based method.

**Figure 3:**
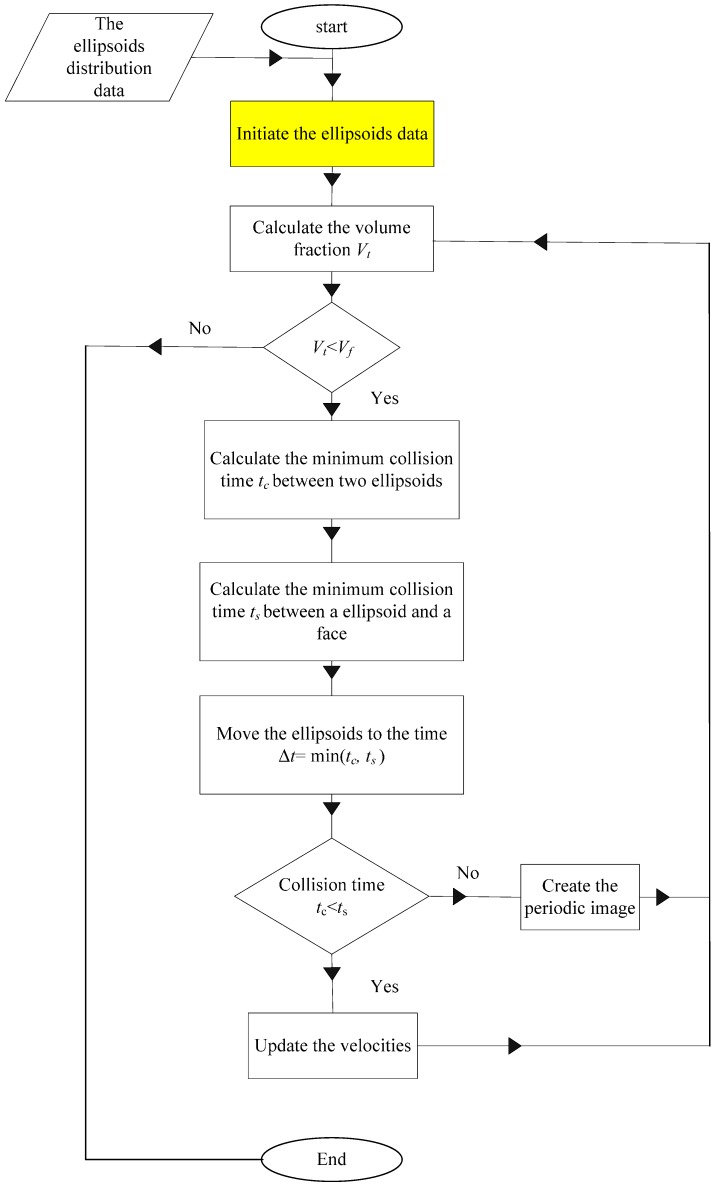
Flowchart of the generation of the periodic random packing of ellipsoids.

All of the centroids of the *N* ellipsoids are randomly created in a cell of side *L* at the beginning. Each ellipsoid has a random orientation and initially a null volume. The ellipsoidal volumes are gradually increasing at a constant growth rate. The growth rates of the semi-principal axes (*a*_0_, *b*_0_, *c*_0_) are chosen so that *a*_0_/*a* = *b*_0_/*b* = *c*_0_/*c*, where (*a*, *b*, *c*) are the semi-principal axes’ lengths of the ellipsoids. At the same time, each ellipsoid has a random linear and angular velocity. The ellipsoids are then set in the translational and rotational motion, and the volumes gradually increase. At each increment, two types of collisions need to be checked, which are the collision between two ellipsoids and the collision between an ellipsoid and a cell face. If the collision between two ellipsoids occurs, the linear and angular velocities of the involved ellipsoids are updated according to the law of the conservation of momentum. If the collision between an ellipsoid and a cell face occurs, the periodic images of the involved ellipsoid are created on the opposite face, line or vertex. The process stops until the volume fraction *V_f_* is reached. The flowchart of the MD-based generation procedure of the periodic random packing of ellipsoids is shown in Figure 3.

### 3.2. Representation of an Ellipsoid

An ellipsoid can be represented by the centroid vector ***r*** and the orientation denoted by two Euler angles, the dip angle *θ* and dip direction *φ*. The quaternion [44] is more convenient and stable than the two Euler angles in numerical calculations. The quaternion of the ellipsoid consists of a scalar and a vector. The vector is the rotation axis of the ellipsoid, and the scalar is the rotation angle. The quaternion of the ellipsoid *i* at time *t* can be written as:

(21) $$q_{t}^{i}=[\alpha_{t}^{i},\psi_{t}^{i}]$$

where:

(22) $$\alpha_{t}^{i}=\cos\frac{1}{2}(\frac{\pi }{2}-\theta_{t}^{i})\cdot \cos\frac{1}{2}\varphi_{t}^{i}$$

(23) $$\psi_{t}^{i}=[-\sin\frac{1}{2}(\frac{\pi }{2}-\theta_{t}^{i})\cdot \sin\frac{1}{2}\varphi_{t}^{i},\sin\frac{1}{2}(\frac{\pi }{2}-\theta_{t}^{i})\cdot \cos\frac{1}{2}\varphi_{t}^{i},\cos\frac{1}{2}(\frac{\pi }{2}-\theta_{t}^{i})\cdot \sin\frac{1}{2}\varphi_{t}^{i}]$$

At the end of the procedure, if ellipsoid *i* at time *t* has the quaternion as Equation (21), the two Euler angles *θ* and φ are given by:

(24) $$\theta_{t}^{i}=\frac{\pi }{2}-\sin^{-1}(2(\alpha_{t}^{i}\psi_{2t}^{i}-\psi_{1t}^{i}\psi_{3t}^{i}))$$

(25) $$\varphi_{t}^{i}=\tan^{-1}\frac{2(\alpha_{t}^{i}\psi_{3t}^{i}+\psi_{1t}^{i}\psi_{2t}^{i})}{1-2({\left(\psi_{2t}^{i}\right)}^{2}+{\left(\psi_{3t}^{i}\right)}^{2})}$$

where $\psi_{kt}^{i}$ (*k* = 1, 2, 3) is the *k*-th term of the vector $\psi_{t}^{i}$.

Assume that the global coordinate system is *Ox*_1_*x*_2_*x*_3_, and the local coordinate system *O’x’*_1_*x’*_2_*x’*_3_ is aligned along the principal axes of ellipsoid *i*. The vector $r_{t}^{i}$ denotes the position of the centroid *O’* at time *t.* The equation of ellipsoid *i* at time *t* in its local coordinate system can be written as:

(26) $${x^{\prime }}^{T}{A^{\prime }}_{t}^{i}x^{\prime }=0$$

where:

(27) $${A^{\prime }}_{t}^{i}=\mathrm{diag}(a_{i}^{t},b_{i}^{t},c_{i}^{t},-1)$$

is a diagonal matrix where $a_{i}^{t}$, $b_{i}^{t}$, $c_{i}^{t}$ denote the length of the semi-principal axes of ellipsoid *i* at time *t*; $x^{\prime }={\left(x\prime_{1},x\prime_{2},x\prime_{3},\omega \prime \right)}^{T}$ are the homogeneous coordinates of a point, which are more convenient in the computation than the Cartesian coordinates. The equivalent Cartesian coordinates of ***x***’ are $x^{\prime }={\left(\frac{x\prime_{1}}{\omega \prime },\frac{x\prime_{2}}{\omega \prime },\frac{x\prime_{3}}{\omega \prime }\right)}^{T}$.

In homogeneous coordinates, the transition from the local coordinates system to the global coordinates system is directly combined by the rotation and translation, i.e.,

(28) $$x=M_{t}^{i}x\prime =\left[\begin{array}{cc} R_{t}^{i} & r_{t}^{i}\\ 0 & 1 \end{array}\right]x\prime$$

where the transition matrix $M_{t}^{i}$ can be obtained from the quaternion of ellipsoid *i* at time *t*. $R_{t}^{i}$ is the rotation matrix given by:

(29) $$R_{t}^{i}=(2{\left(\alpha_{t}^{i}\right)}^{2}-1)I+2\psi_{t}^{i}\psi_{t}^{iT}+2\alpha_{t}^{i}S_{t}^{i}$$

where **I** is the identity matrix and $S_{t}^{i}$ is an antisymmetric matrix given by [44]:

(30) $$S_{t}^{i}=\left[\begin{array}{ccc} 0 & -\psi_{3t}^{i} & \psi_{2t}^{i}\\ \psi_{3t}^{i} & 0 & -\psi_{1t}^{i}\\ -\psi_{2t}^{i} & \psi_{1t}^{i} & 0 \end{array}\right]$$

Substituting Equations (28)–(30) into Equation (26), the equation of ellipsoid *i* at time *t* in the global system becomes:

(31) $$x^{T}A_{t}^{i}x=0$$

where:

(32) $$A_{t}^{i}={\left(M_{t}^{i}\right)}^{-T}{A^{\prime }}_{t}^{i}{\left(M_{t}^{i}\right)}^{-1}$$

${\left(M_{t}^{i}\right)}^{-1}$ is the inverse of $M_{t}^{i}$; ${\left(M_{t}^{i}\right)}^{-T}$ is the inverse of ${\left(M_{t}^{i}\right)}^{T}$.

### 3.3. Moving and Growing an Ellipsoid

The configuration of ellipsoid *i* at time *t* is determined by the position vector $r_{t}^{i}$, the quaternion $q_{t}^{i}$ and the semi-principal axes’ lengths $\left(a_{t}^{i},b_{t}^{i},c_{t}^{i}\right)$. The ellipsoid *i* has a linear velocity ***ν****^i^* and rotation velocity ***ω****^i^*. The growth rates of the ellipsoid *i* are $\left(a_{0}^{i},b_{0}^{i},c_{0}^{i}\right)$. The configuration of ellipsoid *i* at time *t +* Δ*t* can be derived according to the moving and growing rates of the ellipsoid *i.* The semi-principal axes’ lengths at time *t +* Δ*t* are:

(33) $$\left(a_{t+\Delta t}^{i},b_{t+\Delta t}^{i},c_{t+\Delta t}^{i})=(a_{t}^{i}+a_{0}^{i}\Delta t,b_{t}^{i}+b_{0}^{i}\Delta t,c_{t}^{i}+c_{0}^{i}\Delta t\right)$$

The new position of the centroid at time *t +* Δ*t* can be derived by the linear velocity, given by:

(34) $$r_{t+\Delta t}^{i}=r_{t}^{i}+\nu^{i}\Delta t$$

From time *t* to time *t +* Δ*t*, the ellipsoid *i* has been rotated by an angle $\left\|\omega^{i}\right\|\Delta t$ about the unit vector $\frac{\omega^{i}}{\left\|\omega^{i}\right\|}$. This motion can be expressed in terms of a quaternion $q_{\Delta t}^{i}$, given by:

(35) $$q_{\Delta t}^{i}=[\alpha_{\Delta t}^{i},\psi_{\Delta t}^{i}]=[\cos(\frac{\left\|\omega^{i}\right\|\Delta t}{2}),\sin(\frac{\left\|\omega^{i}\right\|\Delta t}{2})\frac{\omega^{i}}{\left\|\omega^{i}\right\|}]$$

The quaternion at time *t +* Δ*t* can be obtained by combining the quaternion $q_{t}^{i}$ and $q_{\Delta t}^{i}$, such that [44]:

(36) $$q_{t+\Delta t}^{i}=[\alpha_{t+\Delta t}^{i},\psi_{t+\Delta t}^{i}]$$

where:

(37) $$\alpha_{t+\Delta t}^{i}=\alpha_{t}^{i}\alpha_{\Delta t}^{i}-{\left(\psi_{t}^{i}\right)}^{T}\psi_{\Delta t}^{i}$$

(38) $$\psi_{t+\Delta t}^{i}=\alpha_{t}^{i}\psi_{\Delta t}^{i}+\alpha_{\Delta t}^{i}\psi_{t}^{i}-\psi_{t}^{i}\times \psi_{\Delta t}^{i}$$

By knowing the position vector $r_{t+\Delta t}^{i}$, the quaternion $q_{t+\Delta t}^{i}$ and the semi-principal axes’ lengths $\left(a_{t+\Delta t}^{i},b_{t+\Delta t}^{i},c_{t+\Delta t}^{i}\right)$, the coefficient matrix ${A^{\prime }}_{t+\Delta t}^{i}$ can be deduced from Equation (27), and the transition matrix $M_{t+\Delta t}^{i}$ is obtained from Equations (29) and (30). Finally, the equation of ellipsoid *i* at time *t +* Δ*t* is given by Equations (31) and (32).

### 3.4. Calculating the Collision Time

#### 3.4.1. Collision Time between Two Ellipsoids

The algebraic condition for the configuration relationship of two ellipsoids *i* and *j* can be represented by the roots of the characteristic equation of the two ellipsoids [45]. The characteristic equation at time *t* can be written as:

(39) $$\det(\lambda A_{t}^{i}+A_{t}^{j})=0$$

where $A_{t}^{i}$ and $A_{t}^{j}$ are the coefficient matrix of ellipsoids *i* and *j*, respectively. Equation (39) is a fourth order polynomial of λ and has four roots. The relationship between the four roots and the configuration of the two ellipsoids is shown in Table 1. The collision time between two ellipsoids is the time *t* + *t_c_* when Condition 2 in Table 1 is satisfied. This is an optimization problem, which can be solved with the bisection method and the secant method.

Once the collision time *t_c_* is obtained, the contact point ***x***_c_ of the two ellipsoid can be solved from the following equation [46]:

(40) $$(\lambda_{0}A_{t+t_{c}}^{i}-A_{t+t_{c}}^{j})x_{c}=0$$

where *λ*_0_ is the double positive root of the characteristic Equation (39) at time *t* + *t_c_*.

#### 3.4.2. Collision Time between an Ellipsoid and Cell Faces

The point coordinates ***x*** in the equation $x^{T}A_{t}^{i}x=0$ of the ellipsoid *i* are a quaternion, but the point coordinates in the equation of a cell face are a vector. Therefore, the homogeneous coordinates need to be transformed to Cartesian coordinates. The coefficient matrix $A_{t}^{i}$ can be rewritten as:

(41) $$A_{t}^{i}=\left[\begin{array}{cc} B_{t}^{i} & \frac{1}{2}d_{t}^{i}\\ \frac{1}{2}{\left(d_{t}^{i}\right)}^{T} & F_{t}^{i} \end{array}\right]$$

where $B_{t}^{i}$ is a 3 × 3 matrix; $d_{t}^{i}$ is a 3 × 1 vector; $F_{t}^{i}$ is a scalar.

The equation of ellipsoid *i* becomes:

(42) $$x^{T}B_{t}^{i}x+d_{t}^{i}x+F_{t}^{i}=0$$

**Figure 4:**
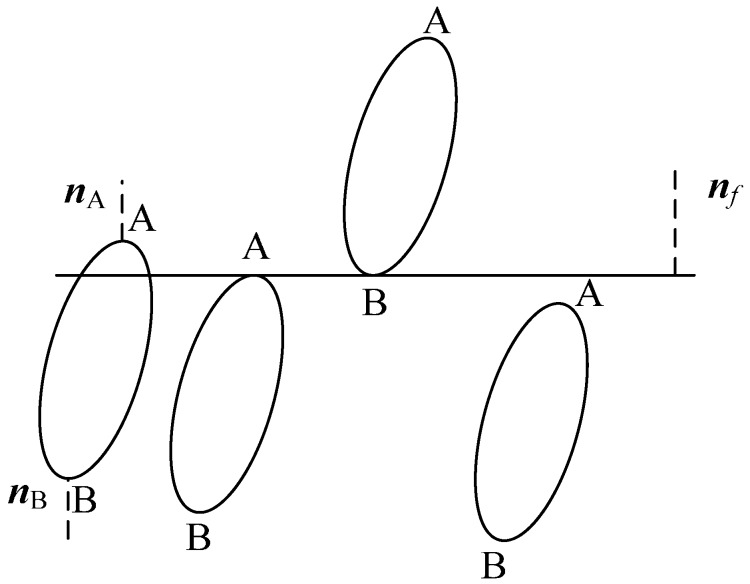
Sketch of the intersection of an ellipsoid with a plane.

The intersection of ellipsoid *i* with a plane ***x****_k_* = *β* (*k* ∈ {1, 2, 3}; *β* ∈ {0, *L*}) is an ellipse, a point or the empty set, which is shown in Figure 4. The intersection type depends on the spatial relationship of the plane with Points A and B, where the normal of the ellipsoid is parallel with the normal of the plane. The normal of the ellipsoid can be derived from the gradient of the ellipsoid equation, such that:

(43) $$n(x)=\nabla (x^{T}B_{t}^{i}x+d_{t}^{i}x+F_{t}^{i})=2B_{t}^{i}x+d_{t}^{i}$$

where n(x) is the normal of the ellipsoid at point ***x***.

Since the normals ***n***_A_ and ***n***_B_ are parallel to the normal of the plane ***n****_f_*, the points ***x***_A_ and ***x***_B_ can be solved from the following equation:

(44) $$n(x)\times n_{f}=(2B_{t}^{i}x+d_{t}^{i})\times n_{f}=0$$

Assuming that ***x***_A*k*_ < ***x***_B*k*_, the intersection relationship of the ellipsoid *i* with the plane ***x****_k_* = *β* is shown in Table 2. The collision time between the ellipsoids *i* with a plane is the time *t* + *t_s_* when Condition 3 in Table 2 is satisfied. This is an optimization problem that can be solved with the bisection method and the secant method.

### 3.5. Post-Processing after the Collision

#### 3.5.1. Update the Velocities

**Figure 5:**
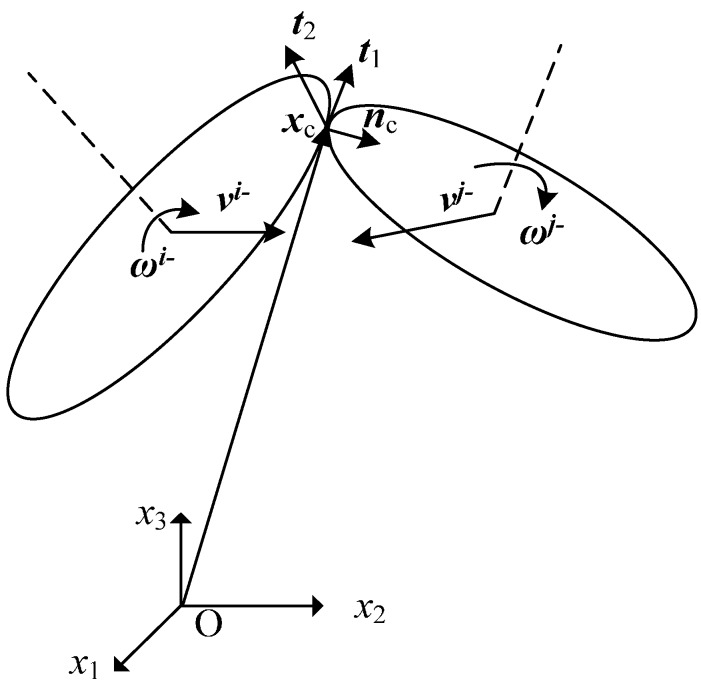
Sketch of the collision of two ellipsoids.

Assuming that the linear and angular velocities of the ellipsoids *i* and *j* before the collision are (***ν****^i−^*, ***ω****^i−^*) and (***ν****^j−^*, ***ω****^j−^*), the linear (***ν****^i+^*, ***ν****^j+^*) and angular (***ω****^i+^*, ***ω****^j+^*) velocities after the collision can be solved according to the law of the conservation of momentum. The friction and the energy loss are assumed negligible. An orthonormal basis (***n***_c_, ***t***_1_, ***t***_2_) is defined at the contact point ***x***_c_, which is shown in Figure 5. ***n***_c_ is the normal of ellipsoid *i* at contact point ***x***_c_, which is defined as Equation (43). The unit vector along ***n***_c_ is given by:

(45) $$n_{c}=\frac{2B_{t}^{i}x_{c}+d_{t}^{i}}{\left\|2B_{t}^{i}x_{c}+d_{t}^{i}\right\|}$$

The unit vectors ***t***_1_ and ***t***_2_ are defined such that (***n***_c_, ***t***_1_, ***t***_2_) form a orthonormal basis, given by:

(46) $$t_{2}=n_{c}\times \frac{x_{c}}{\left\|x_{c}\right\|}$$

(47) $$t_{1}=t_{2}\times n_{c}$$

The linear momentum along the vectors (***n***_c_, ***t***_1_, ***t***_2_) and the angular momentum about the contact point ***x***_c_ are conserved during the collision. The conservation of the linear momentum allows us to write the following equations:

(48) $$m_{t}^{r}{\left(v^{r+}\right)}^{T}t_{1}=m_{t}^{r}{\left(v^{r-}\right)}^{T}t_{1}$$

(49) $$m_{t}^{r}{\left(v^{r+}\right)}^{T}t_{2}=m_{t}^{r}{\left(v^{r-}\right)}^{T}t_{2}$$

(50) $$m_{t}^{i}{\left(v^{i+}\right)}^{T}n_{c}+m_{t}^{j}{\left(v^{j+}\right)}^{T}n_{c}=m_{t}^{i}{\left(v^{i-}\right)}^{T}n_{c}+m_{t}^{j}{\left(v^{j-}\right)}^{T}n_{c}$$

where *r* ∈ {*i*, *j*}; $m_{t}^{r}$ is the mass of the ellipsoid *r* at time *t* with a unit density, which is given by:

(51) $$m_{t}^{r}=\frac{4}{3}\pi a_{t}^{r}b_{t}^{r}c_{t}^{r}$$

Considering the effect of the ellipsoids growing rate, the closing velocity of the two ellipsoids along vector ***n***_c_ must satisfy the following equation in order to avoid the ellipsoids from separating after the collision.

(52) $${\left(\nu^{c+}\right)}^{T}n_{c}=-{\left(\nu^{c-}\right)}^{T}n_{c}-2[\max(a_{0}^{i},b_{0}^{i},c_{0}^{i})+\max(a_{0}^{j},b_{0}^{j},c_{0}^{j})]$$

where ***ν****^c−^* and ***ν****^c+^* are the closing velocities from ellipsoid *i* to *j* before and after the collision, respectively, given by:

(53) $$\nu^{c-}=[\nu^{i-}+\omega^{i-}\times (x_{c}-r_{t}^{i})]n_{c}-[\nu^{j-}+\omega^{j-}\times (x_{c}-r_{t}^{j})]n_{c}$$

(54) $$\nu^{c+}=[\nu^{i+}+\omega^{i+}\times (x_{c}-r_{t}^{i})]n_{c}-[\nu^{j+}+\omega^{j+}\times (x_{c}-r_{t}^{j})]n_{c}$$

The conservation of the angular momentum about the contact point yields the following equation:

(55) $$H_{t}^{r}\omega^{r+}+m_{t}^{r}[(r_{t}^{r}-x_{c})\times \nu^{r+}]=H_{t}^{r}\omega^{r-}+m_{t}^{r}[(r_{t}^{r}-x_{c})\times \nu^{r-}]$$

where *r* ∈ {*i*, *j*}; $H_{t}^{r}$ is the moment of inertia of the ellipsoid *r* at time *t* with a unit density, which is given by:

(56) $$H_{t}^{r}=R_{t}^{r}{H^{\prime }}_{t}^{r}{\left(R_{t}^{r}\right)}^{T}$$

where $R_{t}^{r}$ is the rotation matrix, which is defined as Equation (29); ${H^{\prime }}_{t}^{r}$ is the moment of inertia of ellipsoid *r* in its local coordinate system, given by:

(57) $${H^{\prime }}_{t}^{r}=\frac{m_{t}^{r}}{5}\mathrm{diag}({\left(b_{t}^{r}\right)}^{2}+{\left(c_{t}^{r}\right)}^{2},{\left(a_{t}^{r}\right)}^{2}+{\left(c_{t}^{r}\right)}^{2},{\left(a_{t}^{r}\right)}^{2}+{\left(b_{t}^{r}\right)}^{2})$$

The velocities after the collision (***ν****^i+^*, ***ν****^j+^*, ***ω****^i+^*, ***ω****^j+^*) can be obtained from the solution of Equations (48)–(50), (52)–(55).

#### 3.5.2. Create the Periodic Images

**Figure 6:**
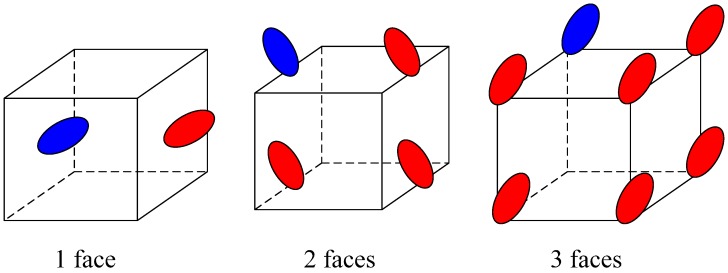
The periodic images when the ellipsoid intersect with different cell faces.

When an ellipsoid intersects with the cell faces at time *t*_s_, the periodic images depend on the number of faces that intersect the ellipsoid. When the number of faces is 1, 2, 3, the number of the periodic images is 1, 4, 7, respectively, which is shown in Figure 6. The periodic images have the same quaternions, velocities and growing rates as the ellipsoid intersecting with the cell faces. The centroid of the periodic images has an offset {−*L*, *L*}, which depends on the face for which the periodic images appear.

### 3.6. RVE Samples

**Figure 7:**
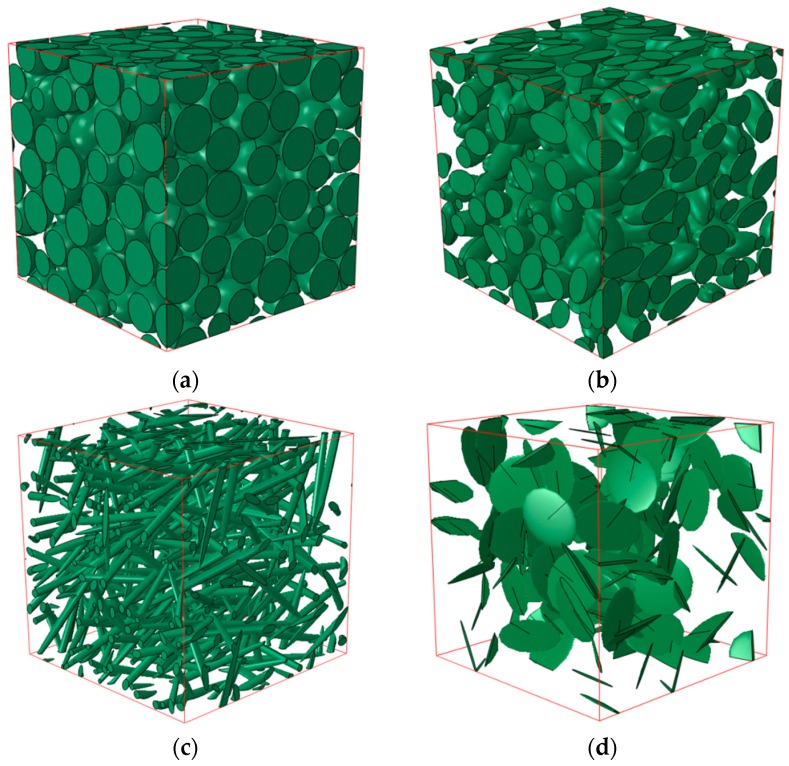
Representative volume element (RVE) samples of different aspect ratios and volume fractions. (**a**) *R*_1_ = *R*_2_ = 1, *N* = 200, *V_f_* = 60%; (**b**) *R*_1_ = *R*_2_ = 2, *N* = 200, *V_f_* = 40%; (**c**) *R*_1_ = *R*_2_ = 20, *N* = 200, *V_f_* = 10%; (**d**) *R*_1_ = *R*_2_ = 1/20, *N* = 100, *V_f_* = 5%

**Figure 8:**
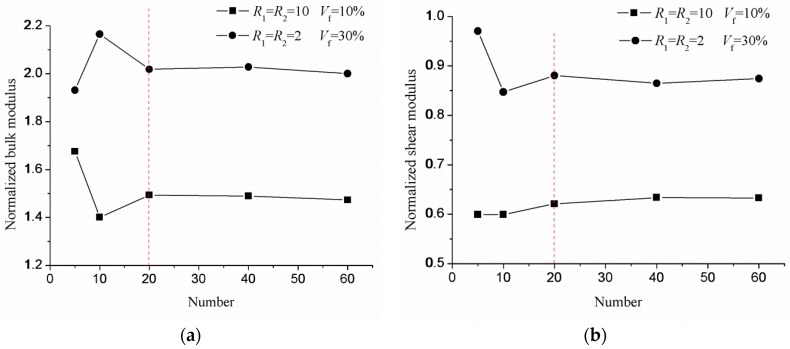
The variation of the normalized modulus with the number of inclusions for the volume fraction being fixed: (**a**) bulk modulus; (**b**) shear modulus.

Four RVE samples of different aspect ratios and volume fractions are generated with the MD-based method presented above, which is shown in Figure 7. Figure 7a–d shows the random packing of the sphere, normal ellipsoid, prolate ellipsoid and oblate ellipsoid, respectively. The aspect ratios are denoted as *R*_1_ = *a*/*b* and *R*_1_ = *a*/*c*, where semi-principal axis *a* is the axis of revolution. The number of inclusions denoted as *N* is the equivalent of the size of inclusions, when the size of the cell is fixed. During the numerical experiments, we tested increasingly larger samples of the material, while keeping the size of the cell fixed. It was found that the effective properties stabilized when approximately 20 inclusions were used in a sample, which will be discussed in Section 4; see Figure 8. Therefore, the samples in Figure 7 satisfy the assumptions of computational homogenization.

## 4. Effective Properties of Composite Materials

We now want to determine the effective elastic properties of the composite material consisting of the periodic random packing of ellipsoidal inclusions embedded in an isotropic elastic matrix. The size, aspect ratio and volume fraction of the ellipsoids will affect the effective properties of the composite material. These parameters are controlled with the RVE generation algorithm. The effective properties of the composite are calculated by computational homogenization as presented in Section 2. The results depend on the mechanical parameters of the inclusions and matrix. The Lame constants of the inclusions and matrix are (*λ*_1_, *μ*_1_) = (1, 1) and (*λ*_2_, *μ*_2_) = (10, 10), respectively. All of the RVEs in this section are assumed as the unit cell and having the normalized Lame constants; thus, the output is the normalized value of the homogenized parameters. The normalized bulk modulus $\frac{\bar{K}}{K_{0}}$ and shear modulus $\frac{\bar{G}}{G_{0}}$ are chosen as the output, where $\bar{K}$, $\bar{G}$ are the homogenized modulus and *K*_0_, *G*_0_ are the modulus of the matrix.

### 4.1. Influence of the RVE Size

Firstly, the size of the representative volume element (RVE) needs to be determined. The size is acceptable when the effective properties of composite materials stabilize if the size is increasing. Since the cells are the unit in all of the RVEs, the RVE size is equivalent to determine the minimal number of ellipsoids, which is sufficiently large to include a sampling of the microstructure and is homogeneous in the sense of macroscopic effective properties. Two cases are considered to determine the minimal number of the ellipsoids. In the first case, the aspect ratios are *R*_1_ = *R*_2_ = 2, and the volume fraction is *V_f_* = 30%. In the second case, the aspect ratios are *R*_1_ = *R*_2_ = 10, and the volume fraction is *V_f_* = 10%. Five numbers *N* (5, 10, 20, 40, 60) are studied for each case. The normalized bulk and shear modulus are shown in Figure 8, which shows that the effective properties stabilize when the number of inclusions exceeds 20. The number of 20 is valid when the aspect ratio *R*_1_, *R*_2_ ≤ 10. Indeed, it is necessary to generate more inclusions to obtain a uniform distribution of ellipsoid orientations when the aspect ratios are higher. This is shown in Figure 8b. The inclusion number of 20 will be considered in all of the following computations.

### 4.2. Influence of the Aspect Ratio

**Figure 9:**
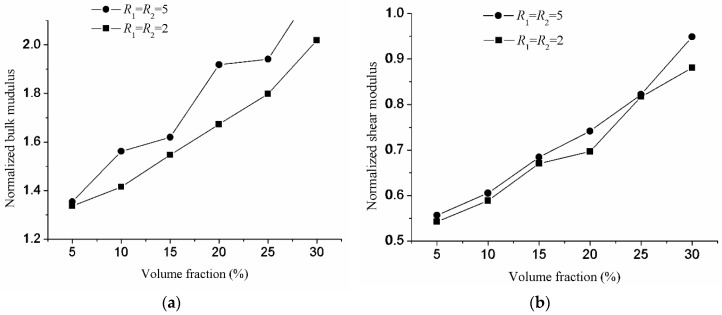
The variation of normalized modulus with the volume fraction for the aspect ratio being fixed: (**a**) bulk modulus; (**b**) shear modulus.

**Figure 10:**
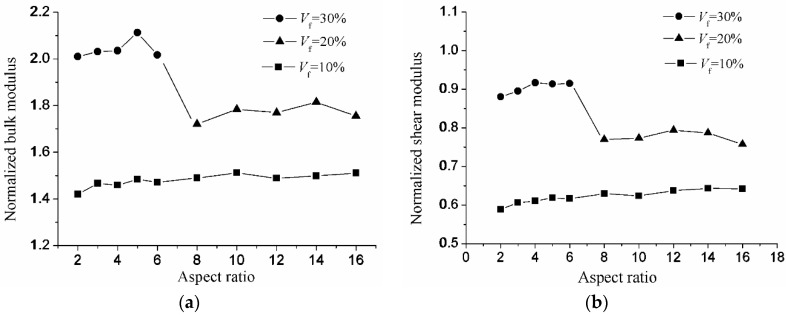
The variation of normalized modulus with the aspect ratio for the volume fraction being fixed: (**a**) bulk modulus; (**b**) shear modulus.

After the minimal number of the inclusions is determined, the influence of the aspect ratio on the effective properties of composite materials will be considered. As we know, the reinforcement effect mainly depends on the volume fraction when the macroscopic properties of the composite materials are isotropic. However, the aspect ratio will affect the reinforcement effect with different volume fractions. Two series of tests will be considered to study the influence of the aspect ratio on the reinforcement effect. Two cases are considered in the first series of the tests. In the first case, the aspect ratios are *R*_1_ = *R*_2_ = 2, and the inclusion number is *N* = 20. In the second case, the aspect ratios are *R*_1_ = *R*_2_ = 5, and the inclusion number is *N* = 20. Four volume fractions *V_f_* (5%, 10%, 15%, 20%, 25%, 30%) are studied for each case. The normalized bulk and shear modulus of the first series of test are shown in Figure 9. Three cases are also considered in the second series of tests. In the first case, the volume fraction is *V_f_* = 10%. In the second case, the volume fraction is *V_f_* = 20%. In the third case, the volume fraction is *V_f_* = 30%. Since the max volume fraction for the aspect ratios *R*_1_ = *R*_2_ > 10 cannot reach 30%, so the aspect ratios *R*_1_ = *R*_2_ (2, 3, 4, 5, 6) are studied for the volume fractions 10% and 30%. Additionally, the aspect ratios *R*_1_ = *R*_2_ (8, 10, 12, 14, 16) are studied for the volume fractions 10% and 20%. For the aspect ratios *R*_1_ = *R*_2_ (2, 3, 4, 5, 6) and (8, 10, 12, 14, 16), the inclusion numbers are *N* = 20 and *N* = 60, respectively. The normalized bulk and shear modulus of the second series of tests are shown in Figure 10.

It is shown in Figure 9 that the dependence of the effective properties of the composite materials on the volume fraction of the inclusions for different aspect ratios is similar. The normalized bulk and shear modulus increase with the increasing volume fraction from 5%–30%. In Figure 9a, it is shown that the normalized bulk modulus is slightly higher under the higher aspect ratios *R*_1_ = *R*_2_ = 5. However, there are no significant variations in the normalized shear modulus with different aspect ratios in Figure 9b. However, the composite materials are better reinforced with a higher aspect ratio with an increasing aspect ratio from 1–5 particularly for *V_f_* = 30%. The influence of the aspect ratio on elastic properties has been studied in GP Tandon’s paper [31]. It is observed that the Young’s modulus and shear modulus increase with the increasing aspect ratio, whereas the bulk modulus decreases with it. These phenomena occur in the plane strain problem. The influence of the aspect ratio on elastic properties in the 3D microstructure is different. From Figure 10, it can be clearly observed that with the increase of the aspect ratio of the inclusions, there are no significant variations in the normalized shear modulus. On the other hand, the normalized bulk modulus increases slightly with increasing aspect ratio from 1–10. After 10, the influence of the aspect ratio gradually decreases. It also can be observed that when the volume fraction decreases from *V_f_* = 30% to *V_f_* = 20%, the normalized bulk and shear modulus significantly decreased. Generally speaking, the influence of the volume fraction on the elastic properties is greater than that of the aspect ratios.

## 5. Conclusions

In this paper, a computational homogenization model has been developed for the evaluation of the effective properties of the composite materials. The general steps of the computational homogenization model have been implemented in ABAQUS. The composite materials have the microstructure of the periodic random packing of ellipsoid inclusions, which are generated with an MD-based method. Different shapes of inclusions are generated in the RVE samples, which show that the MD-based method can generate various kinds of reinforcements and a high volume fraction of inclusions. The influence of the size, aspect ratio and volume fraction of the inclusions on mechanical properties has been studied. These studies showed that the effective properties of composite materials will be stable when the number of the inclusions is high enough to ensure the isotropy of the materials. There is no significant influence on the normalized shear modulus with the increase of the aspect ratio when the volume fraction is fixed. However, for the normalized bulk modulus, the reinforcement effect varies considerably for a lower aspect ratio and stabilizes for a higher aspect ratio. It shows that the MD-based method and the computational homogenization method are efficient tools to evaluate the effective properties of composite materials. In future work, it is desirable to apply the MD-based method to generating the microstructure of rock mass joints and microcracks. Not only modeling the elastic properties, the developed modeling scheme can be extended for the modeling of other effective macroscopic properties, such as effective thermal conductivity and permeability [47].

## Figures and Tables

**Table 1:** Conditions for the configuration relationship of two ellipsoids.

Cases	Conditions	Ellipsoids Configuration
1	Two negative and two positive roots	Separate
2	Two negative and a double positive roots	Externally tangent
3	All other cases	Overlap

**Table 2:** Conditions for the intersection relationship of an ellipsoid with a plane.

Cases	Conditions	Ellipsoids Configuration
1	***x***_A*k*_ < *β* < ***x***_B*k*_	Overlap
2	*β* < ***x***>_A*k*_ or *β* > ***x***_B*k*_	Separate
3	*β* = ***x***_A*k*_ or *β* = ***x***_B*k*_	Tangent
